# Chronic cerebrospinal venous insufficiency in patients with multiple sclerosis

**DOI:** 10.1136/jnnp.2008.157164

**Published:** 2008-12-05

**Authors:** P Zamboni, R Galeotti, E Menegatti, A M Malagoni, G Tacconi, S Dall’Ara, I Bartolomei, F Salvi

**Affiliations:** 1 Vascular Diseases Center, University of Ferrara, Ferrara, Italy; 2 Department of Neurology, Bellaria Hospital, Bologna, Italy

## Abstract

**Background:**

The extracranial venous outflow routes in clinically defined multiple sclerosis (CDMS) have not previously been investigated.

**Methods:**

Sixty-five patients affected by CDMS, and 235 controls composed, respectively, of healthy subjects, healthy subjects older than CDMS patients, patients affected by other neurological diseases and older controls not affected by neurological diseases but scheduled for venography (HAV-C) blindly underwent a combined transcranial and extracranial colour-Doppler high-resolution examination (TCCS-ECD) aimed at detecting at least two of five parameters of anomalous venous outflow. According to the TCCS-ECD screening, patients and HAV-C further underwent selective venography of the azygous and jugular venous system with venous pressure measurement.

**Results:**

CDMS and TCCS-ECD venous outflow anomalies were dramatically associated (OR 43, 95% CI 29 to 65, p<0.0001). Subsequently, venography demonstrated in CDMS, and not in controls, the presence of multiple severe extracranial stenosis, affecting the principal cerebrospinal venous segments; this provides a picture of chronic cerebrospinal venous insufficiency (CCSVI) with four different patterns of distribution of stenosis and substitute circle. Moreover, relapsing-remitting and secondary progressive courses were associated with CCSVI patterns significantly different from those of primary progressive (p<0.0001). Finally, the pressure gradient measured across the venous stenosies was slightly but significantly higher.

**Conclusion:**

CDMS is strongly associated with CCSVI, a scenario that has not previously been described, characterised by abnormal venous haemodynamics determined by extracranial multiple venous strictures of unknown origin. The location of venous obstructions plays a key role in determining the clinical course of the disease.

Multiple sclerosis (MS) is an inflammatory demyelinating disease of the central nervous system (CNS) of unknown pathogenesis.^1^ ^2^ MR venography^3^^–^6 and postmortem studies^7^ have demonstrated a topographic correspondence between MS plaques and cerebral venous system. The drainage through the extracranial venous outflow routes has not previously been investigated in MS patients.

Posture and the mechanic movement of respiration play a fundamental role in ensuring the correct cerebrospinal venous outflow.^8^ At the time of expiration, the intrathoracic pressure is approximately −5 cm H_2_O, and inspiration causes a respiratory muscular action that can generate an even lower intrathoracic pressure, −8 cm H_2_O. The resulting gradient favours venous return to the right heart, which can be easily assessed with high-resolution echocolour Doppler (ECD) and transcranial colour-coded Doppler sonography (TCCS), which represent an ideal method by which to investigate the haemodynamics of cerebral venous return.^9^^–^19 In addition, ECD clarified the postural control of the extracranial outflow pathways, as follows:^8^^–^12

- the internal jugular vein (IJV) is the predominant pathway in the supine position, confirmed by an increased cross-sectional area of the internal jugular vein (CSA) related to increased blood volume in that posture; and
- redirection of venous flow to the vertebral veins (VVs) occurs in the upright position, with compliant reduction of the CSA of the IJV.

In contrast, MR and selective injection venography are of course limited in evaluating cerebral venous haemodynamics under different postural and respiratory conditions. However, the latter especially provide excellent morphological but static details.

We present the results of a study that evaluated the abnormalities of the cerebral venous outflow in patients with MS using ECD-TCCS and selective venography.

## METHODS

### Non-invasive screening

#### Patients and controls

We investigated 65 patients affected by clinically defined MS (CDMS) and diagnosed according to the revised McDonald criteria.^20^ This group included 35 patients with a relapsing-remitting (RR) clinical course, 20 with secondary progressive (SP), and 10 with primary progressive (PP) course.^21^ ^22^ A relative expanded disability disease score (EDSS) was attributed to each group.^23^ Detailed data regarding their clinical, demographic, and genetic characteristics, MRI examination, presence of oligoclonal bands in the cerebrospinal fluid (CSF) are provided in table 1. PP patients and 18 out of 55 RR-SP were not under treatment at the time of the evaluation.

**Table 1:** Clinical and demographic characteristics of clinically defined multiple sclerosis (MS) patients

	Whole MS no = 65	MS relapsing-remitting no = 35	MS secondary progressive no = 20	MS primary progressive no = 10
Age (years)	41 (34 to 48)	35 (29 to 41)	45 (42 to 52)	58 (46 to 60)
Sex: percentage male, male/female	46%, 30/35	46%, 16/19	45%, 9/11	50%, 5/5
EDSS	2.5 (1 to 5)	1.5 (0.5 to 2)	5 (3.5 to 6.5)	4.3 (3 to 6.5)
Disease duration (years)	6 (3 to 13)	4 (1 to 7)	13 (6 to 21)	10 (5 to 14)
HLA2 (DR15) haplotype carriers (C)+%+/tot	54% 15/28	71% 10/14	27% 3/11	67% 2/3
Cerebrospinal fluid oligoclonal bands+%+/tot	91% 40/44	86% 19/22	100% 17/17	80% 4/5
Compliance with at least three of four MRI revised McDonald criteria%+/tot	100% 65/65	100% 35/35	100% 20/20	100% 10/10

No significant differences were found among MS subgroups for age, expanded disability disease score (EDSS), or disease duration (ANOVA). HLA2 (DR15) genetic analysis was available in 28/65 patients, and cerebrospinal fluid in 44/65.

As controls, we investigated 235 subjects subdivided as follows:

1. 60 healthy subjects matched for age and gender with MS patients (HM-C).
2. 82 healthy subjects older than the median age of onset of CDMS (HA-C);^21^ had haemodynamic anomalies been present in the second control group, we would not have been able to maintain that they have a role in MS, since the disease is not more expected in this age category.
3. 45 patients affected by other neurological diseases (OND) (table 2); this group was composed of patients affected by neurodegenerative disorders (Parkinson disease and amyotrophic lateral sclerosis-ALS), other neuroimmunological disorders including myasthenia gravis and multifocal motor neuropathy (MMN), and cerebrovascular disease (ischaemic stroke, transient ischaemic attack (TIA)).
4. 48 other controls not affected by neurological diseases (table 2), but scheduled for venography (HAV-C) for other pathologies: diagnostic sampling of the IJVs for primary/secondary hyperparathyroidism, varicocele and/or pelvic congestion syndrome, Cockett and thoracic outlet syndromes, indwelling central venous catheters or pacemaker wires, stenosis of venous access for haemodialysis and removal of temporary cava filters.

**Table 2:** Demographics of the control populations

	Group HM-C (n = 60)	Group HA-C (n = 82)	Group HAV-C (n = 48)	Group OND (n = 45)
Age median (25th to 75th percentile)	37 (28 to 49)	58 (51 to 72)	55 (32 to 70)	60 (51 to 77)
Sex %M M/F	46% 28/32	40% 29/43	56% 27/21	56% 25/20

HA-C, healthy aged controls; HAV-C, older controls not affected by neurological diseases but scheduled for venography; HM-C, healthy controls matched for age and gender with multiple sclerosis patients; OND, controls affected by other neurological diseases.

#### Exclusion criteria

We excluded subjects who had Behçet disease, vasculitis, cerebral vascular malformations and congenital vascular malformations (Klippel–Trenaunay, Parkes–Weber, Servelle–Martorell and Budd–Chiari syndromes).

Patients and controls underwent a non-invasive study of cerebrospinal venous return at the Vascular Lab; the ultrasound technicians and physicians interpreting the data were blinded to the patient diagnostic category.

### Study of cerebrospinal venous drainage

Cerebrospinal venous return was examined with the subject positioned on a tilt bed by combining the extracranial ECD methodology for investigating the IJVs and VVs with that of the TCCS for studying the deep cerebral veins (DCVs).^9^^–^19 We focused in particular on the detection of five parameters, which are absent in normal subjects:

1. reflux in the IJVs and/or VVs in sitting and supine posture;
2. reflux in the DCVs;
3. high-resolution B-mode evidence of IJV stenoses;
4. flow not Doppler-detectable in the IJVs and/or VVs;
5. reverted postural control of the main cerebral venous outflow pathways.

#### Reflux in the IJVs and/or VVs in sitting and supine posture

In normal subjects, flow in the IJVs and VVs is directed toward the heart in any position of the head.^8^^–^15 According to a recent study on reflux time cut-off values, we considered reflux a flow reversal from its physiological direction for a duration of >0.88 s.^13^ Flow was assessed during a short period of apnoea following a normal exhalation,^9^ and never in a forced condition such as the Valsalva manoeuvre.^13^ ^14^

We assessed the presence of reflux with the body positioned respectively at 0° and +90°, in the four extracranial venous drainage pathways.^19^

#### Reflux in the DCVs

Physiological intracranial venous flow is monodirectional.^16^^–^19 TCCS investigation assessed the presence of reflux in at least one of the DCVs (internal cerebral vein, basal vein of Rosenthal, great vein of Galen). Participants were examined in both sitting and supine positions, and the venous flow was elicited by inviting the subject to breathe and setting the reflux time to a value >0.5 s.^18^ ^19^

#### High-resolution B-mode evidence of proximal IJV stenoses

We assessed the presence of stenosing venous imaging by means of a complete ECD high resolution B-mode exploration of the cervical vessels, and measurement of the CSA of the IJV. A CSA ⩽0.3 cm^2^, never measured in normal subjects, was taken as reference value.^15^

#### Flow not Doppler detectable in the IJVs and/or VVs

We assessed the lack of a Doppler detectable venous flow in the IJVs and/or VVs despite numerous deep inspirations, with the head positioned at 0° and +90° in the four extracranial venous drainage pathways. In normal subjects this finding was never observed with the head in any position,^9^ but was reported in the supine position in 6% of cases.^12^

#### Reverted postural control of the main cerebral venous outflow pathway

ΔCSA in the IJVs, obtained by subtracting the CSA measured in the supine from that in the sitting position, is a positive value in normal subjects.^8^^–^11 19 We assessed the occurrence of a negative ΔCSA value, representing the loss of postural control of the predominant outflow route in the supine position.

### ECD-TCCS criteria for venography

Diagnosis of suspicious abnormal extracranial cerebral venous outflow required at least two of the five above listed criteria to be fulfilled, and was taken as an indication to continue the study using selective venography in all suspected subjects.

This study was approved by the Ethics Committee of the Ferrara University Hospital. Our Ethics Committee approved the use of selective venography only in subjects (patients or controls) with abnormal venous ultrasonographic examination. An invasive investigation (potential harmful radiation/catheterisation to healthy subject) was felt unnecessary when the ECD scan was negative at the level of the neck.

We therefore used ultrasonography as a screening to venography. Finally, the Committee approved an additional venographic investigation to be carried out on patients without neurological disease, even if preoperative screening for venous return anomalies was negative but who, for other reasons, should have had a venographic examination (HAV-C group).

## SELECTIVE VENOGRAPHY

Sixty-five subjects with MS fulfilling the ECD-TCCS screening criteria and 48 controls of the HAV-C group underwent selective catheterism of the azygous and IJV system via the transfemoral route. Venography was performed, being aware of patients’ diagnoses. We considered a significant stenosis to be any venous lumen reduction greater than 50%.^24^^–^28 In addition, selective venography allowed us to measure with a manometer the pressure expressed in cm/H_2_O in the superior vena cava, in the azygous vein, and in both the IJVs.

### Statistical analysis

Clinical and demographic data are expressed as the median and 25th–75th percentile, and venous pressure as the mean (SD). Differences among groups were tested for significance with the one-way ANOVA analysis of variance. The two-sided Fisher exact test followed by the determination of odds ratio (95% CI) was used for determining the associated risk of MS in case of positive ultrasonographic findings, by comparing the whole MS group with the control group.

The two-sided Fisher exact test was also used for testing differences in the number of extracranial venous strictures between CDMS patients treated and not treated with drugs. Differences in venous pressure between patients and controls, as well as across the stenosies, were analysed with the Mann–Whitney test.

Finally, the χ^2^ test for independence was used for assessing clinical differences of MS patients among the different patterns of extracranial venous outflow obstruction.

p Values up to 0.05 were considered statistically significant.

## RESULTS

### Non-invasive screening

**Figure 1:**
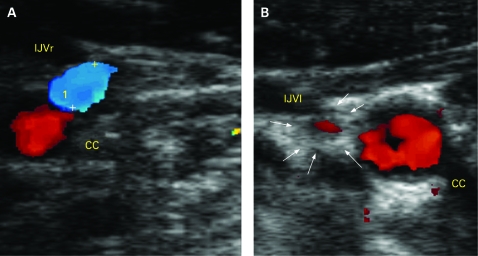
B-mode detection of venous stenosis. (A) Right cervical side, high-resolution B-mode image, transversal access: common carotid artery (CC) with cerebral inflow (red), and right internal jugular vein (IJVr) with regular cerebral outflow (blue). (B) Same patient, left side: stenoses of the left internal jugular vein (IJVl) due to annulus (black arrows) with reflux (red) and severe reduction of the lumen.

Table 3 reports the five TCCS-ECD criteria used for investigating the presence of abnormal extracranial venous outflow, and the relative distribution in RR, SP and PP cases and in controls, followed by OR. None of the controls, including those who had HAV-C, were positive for more than one of the criteria. In MS patients, we found 180 positive criteria and 145 negative criteria (table 3); in contrast, merging all control groups, positive criteria were 33 and negative criteria 1142. Consequently, the risk of MS was dramatically increased by 43-fold (OR 43, 95% CI 29 to 65, p<0.0001), Fisher exact test). Finally, in 37% of cases, B-mode high-resolution imaging allowed directly closed stenosies to be detected in the IJVs (fig 1A,B, table 3).

**Table 3:** Transcranial and extracranial colour-Doppler high-resolution examination (TCCS-ECD) criteria of highly suspected anomalous venous outflow

TCCS-ECD criteria	MS-relapsing-remitting (N; %)	MS-secondary progressive (N; %)	MS-primary progressive (N; %)	Whole MS (N; %)	Control populations (N; %)	Odds ratio all MS vs all controls (95% CI)	p Value
1. Reflux constantly present in IJVs and/or VVs with the head at 0° and +90°	27/35 77%	15/20 75%	4/10 40%	46/65 71%	0/235 0%	1123 (67 to 19 000)	<0.0001
2. Reflux in the deep cerebral veins	19/35 54%	12/20 60%	9/10 90%	40/65 61%	0/235 0%	748 (45 to 12 542)	<0.0001
3. High resolution B-mode evidence of proximal IJV stenoses	9/35 26%	10/20 50%	5/10 50%	24/65 37%	1/235 0%	137 (18 to 1041)	<0.0001
4. Flow not Doppler detectable in the IJVs and/or VVs despite numerous deep inspirations with the head at 0° and +90°	22/35 63%	7/20 35%	5/10 50%	34/65 52%	7/235 3%	36 (15 to 88)	<0.0001
5. Negative ΔCSA in the IJV	18/35 51%	13/20 65%	5/10 50%	36/65 55%	25/235 11%	10 (5 to 20)	<0.0001

OR was calculated for each ultra-sonographic criterion by means of the two-sided Fisher exact test, by comparing the whole MS population with the control group.

CSA, cross-sectional area of the internal jugular vein; IJV, internal jugular vein; MS, multiple sclerosis; VV, vertebral vein.

### Selective venography

**Figure 2:**
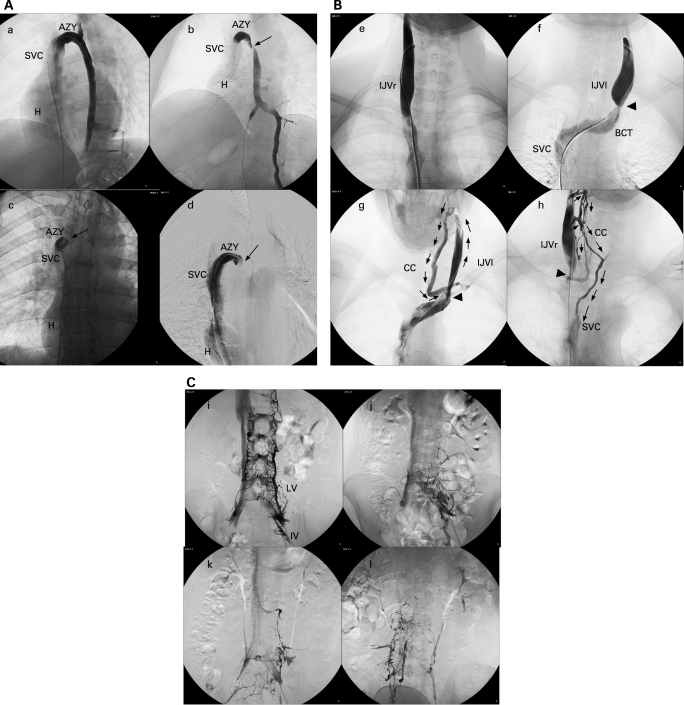
(A) Selective venography of the azygous vein in a control case (HAV-C) (a) and in MS cases (b, c, d). (a) Normal azygous vein, azygous arch and descending trunk (AZY); H, heart; SVC, superior vena cava. (b) Twisting (arrow) just below the azygous arch. (c) Membranous obstruction (arrow) at the junction of the AZY with the SVC. (d) Septum (arrow) of the proximal AZY. (B) Selective venography of the internal jugular vein (IJV) in a control case (HAV-C) (e) and in multiple sclerosis (MS) cases (f, g, h). (e) Normal right IJV (IJVr) with normal outflow and without stenosis after injection. (f) Annulus of the left jugular vein, JVl (IJVl, arrow) at the junction with the brachiocephalic trunk (BCT). (g) Closed stenosis of the IJVl (arrow) with reflux after injection and collateral circles (CC) depicted by small arrows. (h) Annulus of the IJVr (arrow) with reflux and activation of numerous cervical collateral circles involving the thyroid veins (CC); one of them re-enters the SVC. (C) Selective venography of the lumbar veins in a control case (HAV-C) (i) and in MS cases (j, k, l). (i) Selective venography of the ascending lumbar vein (LV) from the iliac vein (IV): normal appearance with characteristic hexagonal shape of the intrarachidian plexus draining outward into the LV and upward to the azygous system. (j, k, l) Dramatic bare venous lumbar tree in MS cases with combination of agenesia and atresia. This picture is further associated with multilevel stenosis of the azygous system configuring the chronic cerebrospinal venous insufficiency type D pattern.

Selective venography demonstrated that the detection of at least 2/5 TCCS-ECD criteria of suspected anomalous extracranial venous outflow (which never occurred in the control populations) was always related to multiple significant extracranial venous stenosis, localised at the cervical, thoracic and, less commonly, abdominal level of the principal cerebrospinal venous segments. In none of the HAV-C subjects who underwent venographic investigation with negative ultrasound were there any stenotic patterns in the IJVs, azygous and lumbar territory (fig 2A (a), B (e), C (i)).

In particular, the azygous vein in the MS group was affected in 86% of cases. Most cases involved membranous obstruction of the junction with the superior vena cava, twisting, or, less frequently, septum and atresia, as can be seen in the *x* rays in fig 2A (b, c, d); in 12 cases the azygous system presented stenoses at several points up to even atresia of the lumbar plexuses (18%) (fig 2C (j, k, l)).

As for the jugular veins, they were found to be stenosed unilaterally or bilaterally in 59/65 patients (91%). The stenoses were frequently annulus and septum, followed by atresia; no twisting was observed (fig 2B (f, g, h)).

Finally, the number of extracranial venous wall stenoses did not differ significantly in patients treated with immunosuppressant/immunomodulator agents or in never-treated patients (p = ns, Fischer exact test).

#### Venous pressure

Pressures measured in patients and controls respectively were not significantly different (Mann–Whitney) (superior vena cava 13 (SD 4) vs 13 (4), azygous 16 (7) vs 14 (4), IJVs 14 (4) vs 12 (5)). In contrast, the pressure gradient measured in CDMS across the stenosies was significantly different. For instance, pressure in the stenotic proximal azygous vein was 3.9 cm/H_2_O higher as compared with the pressure measured in the adjacent superior vena cava of the same subjects (p<0.01; Mann–Whitney); equally, pressure in the stenotic IJVs was 1.8 cm/H_2_O higher with respect to the cava (p<0.04; Mann–Whitney).

#### Patterns of chronic cerebrospinal venous insufficiency

**Figure 3:**
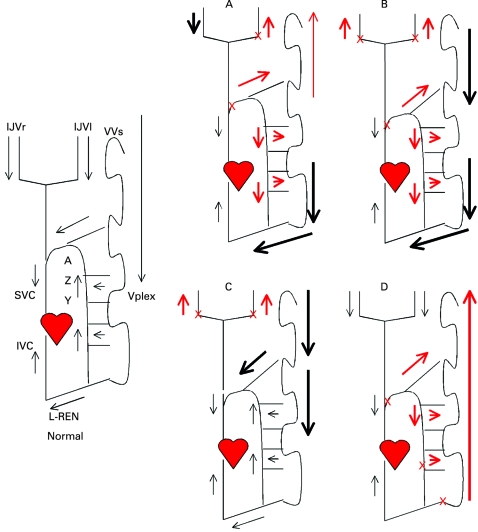
Patterns of chronic cerebrospinal venous insufficiency observed in multiple sclerosis (MS) cases. Normal: example of normal extracranial venous outflow direction. In particular, the black arrows depict the drainage of the internal jugular vein (IJV) system into the superior vena cava (SVC), and of the vertebral plexus (Vplex) outward from the spinal cord into the azygous system (AZY). Type A (30%): this pattern is characterised by a steno-obstruction of the proximal azygous, associated with a closed stenosis of one of the two IJVs (red crosses). Reflux is always present, under all postural conditions, in the stenosed IJV (red arrow), with a compensatory controlateral IJV that appears with an ample cross-sectional area of the IJV. Reflux in the deep cerebral veins (DCVs) was detected by means of transcranial colour-coded Doppler sonography in 60% of cases. In the azygous vein the reflux has an effect as far as the lumbar veins, being able to re-enter the caval circle either through the system of the hemiazygous vein–left renal vein, or by rising again inside the rachis. Type B (38%): this pattern is characterised by significant stenoses of both IJVs and the proximal azygous (red crosses). Reflux is present in all three venous segments (red arrows). Cerebral venous outflow for overcoming the IJVs stenosis re-enters the heart mainly through cervical collateral circles (fig 1B); for the hampered azygous vein outflow, the collateral circles include again the intrarachidian pathway, or the system of the renal-hemiazygous. Type C (14%): this pattern is characterised by bilateral stenosis in both IJVs, with a normal azygous system (red crosses). Reflux (red arrows) occurs in the IJVs but not in the vertebral veins, with cervical or intracranial collateral circles that shunt blood towards the superior vena cava or the azygous vein system, respectively. The resulting overload of the azygous system is depicted by black bold arrows. Type D (18%): in this pattern the azygous system was constantly affected in various segments (red crosses), resulting in a forced venous drainage towards the intrarachidian circles in an upward direction (red arrows). The vertebral veins appeared to be refluent, and the intracranial collateral circles seek to gain the IJVs, as confirmed by reflux detection in DCVs in 90% of cases. At times, the IJVs were also affected (six cases, 50%), causing an additional obstruction in these patients. IVC, inferior vena cava; L-REN, left renal vein.

Selective venography enabled us to localise exactly not only the places of venous steno-obstruction, but also, by comparing the flow direction data collected by the ECD-TCCS method, to identify the pathways of venous reflux and substitute collateral circles. In this way it was possible to delineate a picture of chronic cerebrospinal venous insufficiency (CCSVI) associated with MS, for which we found four principal patterns, as shown in fig 3.

#### Relationship between patterns of CCSVI and clinical course

We also found a highly significant difference in the distribution of the clinical courses among the CCSVI patterns (p<0.0001, χ^2^ test) (table 4). In particular, the location of venous obstruction seems to be a key element influencing the clinical course of the disease. Types A and B correlated with a RR course (83%) with a conversion in the SP course in 70% of cases. In contrast, the PP forms occurred more frequently in the type D pattern (75%).

**Table 4:** Patterns of venous obstruction according to the clinical course

	Relapsing-remitting	Secondary progressive	Primary progressive	p Value, χ^2^ test
Type A	10	5	0	<0.0001
	75%	25%	0%	
Type B	19	9	1	
	66%	31%	3%	
Type C	4	5	0	
	44%	56%	0%	
Type D	2	1	9	
	17%	8%	75%	

## DISCUSSION

In this study we described the association between MS and the altered modality of venous return determined by extracranial multiple venous strictures. In our controls, venography resembled the normal imaging of extracranial cerebrospinal veins.^25^ The hampered cerebrospinal venous drainage in patients with MS determines a complex haemodynamic picture defined as CCSVI. It is characterised by multiple substitute circles, with a very high incidence of reflux in both intracranial and extracranial venous segments, and loss of the postural regulation of cerebral venous outflow.

The mechanism underlying this reflux differs from the reflux caused by incompetence of the jugular valve. In the latter case, valvular insufficiency tested with Valsalva can be related to a picture of transient global amnesia.^14^ In our study, the reflux occurred in any body position without the need to elicit it by a forced movement, suggesting that it is not an expression of valvular incompetence but rather of a stenosing lesion that cannot be crossed with postural or respiratory mechanisms, thereby becoming a long-lasting reverse flow.

Substitute circles are alternative pathways or vicarious venous shunts^29^ (fig 3) that allow for the piping of blood toward available venous segments outside the CNS. In accordance with the pattern of obstruction, both the intracranial and the intrarachidian veins can also become substitute circles; they permit redirection of the deviated flow, preventing intracranial hypertension. However, over time, they become overloaded because they carry two different flows, their own draining flow and the shunted flow (fig 3).

The ECD-TCCS protocol was performed once by a single team of investigators (EM and PZ: vascular technician and interpreting physician, respectively), thus not permitting the assessment of the intraobserver and interobserver variability coefficient. This is a limitation of our study because the assessment of the reproducibility of the proposed protocol, although beyond the aim of the present study, certainly deserves further investigation. However, it should be noted that our ultrasonic assessment can be easily performed in the clinical setting, and, despite the operator-dependency of ultrasounds, there is general agreement on the proposed technique.^9^^–^19

Our results need to address two main questions:

1. Does CCSVI influence the clinical course of MS?
2. Are venous stenoses the cause or products of MS?

First, we identified four main patterns of CCSVI, according to the location, number and association of venous stenosis, and the modality of collateral circulation.

We also observed that the PP course was related to a CCSVI pattern significantly different as compared with RR and SP, suggesting that the location of venous obstruction plays a key role in determining the clinical course.

**Figure 4:**
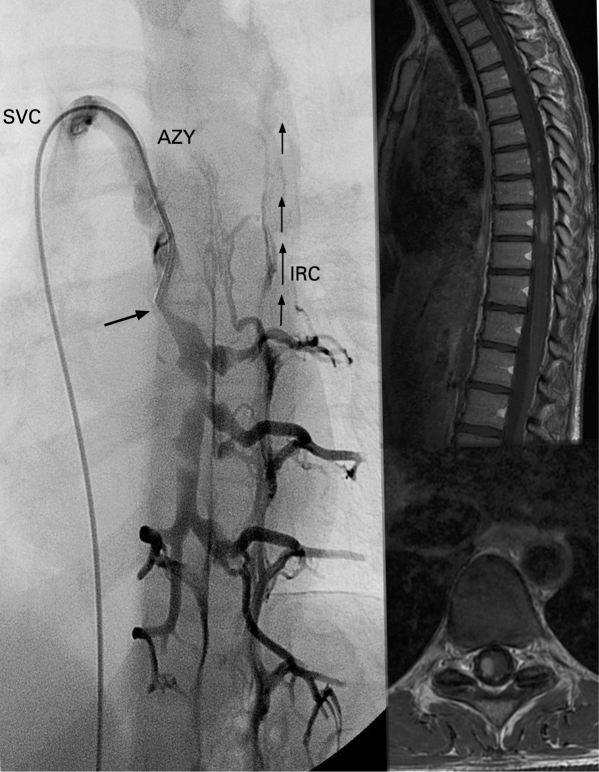
Selective venography and MRI in a clinically defined multiple sclerosis case with chronic cerebrospinal venous insufficiency pattern D. Left: selective venography showing membranous obstruction of the outlet of the azygous vein (AZY) combined with atresia of the descending azygous vein (arrow). Due to multilevel obstruction of the azygous system, the vertebral plexus is dilated below the atresia, and the blood is drained through intrarachidian collateral circles (IRC) in an upward direction. Top right: sagittal T1 weighted imaging after gadolinium injection of the same case, showing typical multiple sclerosis lesions of the spinal cord. The enhanced gadolinium image shows intrarachidian venous plexuses in the form of small spots. Bottom right: axial merge T2 of the same patient at the cervical level with dilated extrarachidian venous plexuses. SVC, superior vena cava.

For instance, PP course, characterised by a slowly progressive syndrome with spastic paraparesis and MRI demonstration of MS plaques in the spinal cord,^20^ ^30^^–^32 was significantly associated with obstruction at several levels of the azygous vein and of the lumbar plexuses (type D pattern, fig 3, table 4). In this situation, venous blood of the spinal cord can be drained only in an upward direction and is shunted toward the venous plexuses inside the spine (figs 3, 4), thus helping to explain the correlation between type D and spinal cord involvement in PP patients.

In contrast, the RR course was significantly associated with type A and B patterns (83% of cases), and particularly to type A, a group in which three-quarters of patients were RR (table 4). The more favourable clinical course could be explained in the latter by the presence of a patent IJV (fig 3), with a compensatory outflow function proved by increased CSA (data not shown). Finally, the conversion to SP course was consistently observed in type A, B, C patterns (95%), but proportionally higher in patients with both the IJVs blocked (56% of type C). However, longitudinal studies are needed, with clinical and advanced MRI analyses^3^ of MS diffusion over time and space in relation to the CCSVI patterns discovered and described in this study.

Second, regarding the causative role of CCSVI in MS, our review of the literature revealed descriptions of associations between the extracranial venous obstructive malformations described herein and disabling neurological pictures however, the latter were defined by these authors generically as myelopathies, without a precise diagnosis and any mention of MS.^27^ ^28^

Venous hypertension has been hypothesised as a cause of MS,^33^ ^34^ but in our study blood pressure was not found to be significantly different from that measured in controls. However, it has been recently demonstrated that a pressure gradient across a central vein stenosis of 2.2 cm/H_2_O corresponds to a CSA reduction greater than 50%.^35^ In our study the pressure gradient across the stenosies between the cava and the azygous arch was significantly different and measured 3.9 cm/H_2_O (fig 2A). The gradient between the stenotic IJVs and the superior vena cava was lower, 1.8 cm/H_2_O, but again significantly different.

Moreover, the absence of Doppler and venographic features of CCSVI in controls suggests that venous obstructions may be causative of MS rather than a coincidental finding.

Interestingly, similar venous stenoses considered to be congenital malformations have been described in other human diseases, that is, membranous obstruction of the inferior vena cava and a minor group of chronic venous diseases of the lower limbs.^27^ ^28^ Such venous obstruction brings about an insufficient venous drainage, respectively, at the level of the liver and of the cutaneous tissue, subsequently causing inflammation, sclerosis, and degenerative lesions.^24^ ^25^ ^36^

In contrast with the malformation hypothesis, cases have been reported of white cell infiltration and endophlebohypertrophy in venous valves where endocarditis is present.^37^

Even though this correlation has never been studied in MS, reports of valvulitis in the course of significant inflammatory disease should certainly be taken into consideration, since they support the hypothesis that these malformations are a result of CDMS rather than a cause. However, if vessel abnormalities were due to an inflammatory-autoimmune disease, they would be less frequent in patients treated with immunomodulating/immunosuppressant agents. On the contrary, our analysis in the RR-SP group did not demonstrate an increased number of extracranial venous stenosing lesions in untreated as compared with treated patients.

Finally, an additional possibility could be related to the side effects of MS drugs on the venous wall, although these have never been reported.^38^

The hypothesis of venous malformations of congenital/developmental origin associated with CDMS seems to be plausible. Nevertheless, additional longitudinal studies are necessary to confirm this hypothesis, as well as to understand the contribution of chronic insufficient venous drainage of the CNS to the process of inflammation and neurodegeneration. Finally, on the basis of our study, we propose the introduction of the ECD-TCCS protocol when a patient presents the first acute episode of demyelinating origin, mostly involving the optic nerve, the so-called clinically isolated syndrome (CIS). Currently, only longitudinal clinical and MRI observation in time and space is capable of establishing the possible conversion of a CIS into a CDMS.^20^
